# Material’s Strength Analysis of the Coupling Node of Axle of the Truck Trailer

**DOI:** 10.3390/ma16093399

**Published:** 2023-04-26

**Authors:** Živilė Decker, Jurijus Tretjakovas, Kazimierz Drozd, Vitalijus Rudzinskas, Mariusz Walczak, Artūras Kilikevičius, Jonas Matijosius, Iryna Boretska

**Affiliations:** 1 Department of Mechanical and Material Engineering, Faculty of Mechanics, Vilnius Gediminas Technical University, J. Basanavičiaus g. 28, LT-03224 Vilnius, Lithuania; zivile.decker@vilniustech.lt (Ž.D.); vitalijus.rudzinskas@vilniustech.lt (V.R.); 2 Department of Applied Mechanics, Faculty of Civil Engineering, Vilnius Gediminas Technical University, Saulėtekio al. 11, LT-10223 Vilnius, Lithuania; jurijus.tretjakovas@vilniustech.lt; 3 Faculty of Mechanical Engineering, Lublin University of Technology, Nadbystrzycka 36, 20-618 Lublin, Poland; k.drozd@pollub.pl; 4 Mechanical Science Institute, Vilnius Gediminas Technical University, J. Basanavičiaus g. 28, LT-03224 Vilnius, Lithuania; arturas.kilikevicius@vilniustech.lt (A.K.); jonas.matijosius@vilniustech.lt (J.M.); 5 Department of Information Technologies, Ukrainian National Forestry University, 103, Gen. Chuprynky, 79057 Lviv, Ukraine; boretska@nltu.edu.ua

**Keywords:** failure analysis, FEM analysis, fracture mechanics, macroscopic research, semi-trailers

## Abstract

Road transport plays an important role in the transport of goods and people and is important for the national economy. Damage usually excludes the means of transport from operation, which causes disruption of supply chains. One such damage is the failure of the suspension system of the vehicle or trailer, which usually occurs when the vehicle is heavily loaded. Such a defective system has been analyzed in this publication. Mathematical apparatus and finite element method (FEM) numerical simulations were used. A dangerous axle cross-section in terms of load was indicated and the maximum stresses in this area were calculated for two types of roads. On highways, the stress at the critical point was 199 MPa, and on uneven roads it increased to 304 MPa, which is comparable to the yield point. It was found that the second form of vibration may cause stresses in the damage area, but the excitation frequency would have to be quite high. The probability of such a load and failure event occurring is low under operating conditions.

## 1. Introduction

Trucks with semi-trailers are the main means of transporting goods by road all over the world. Transport companies have one economic goal—to transport as many goods as possible with the least number of journeys and at the lowest possible cost. In order to meet these expectations, these vehicles must be characterized by high quality construction, durability and reliability. In addition to the construction, suspension, drive and safety systems play an important role. In turn, the reliable operation of these systems has a direct impact on road safety [1,2,3,4,5,6,7,8,9,10]. On the other hand, the safety issues related to the transport of chemical products in road transport are presented in [11,12,13]. The durability of vehicle suspension components refers to the duration of the onset of fatigue, defined as the number of cycles to a specific length of component failure under cyclic loads [6,14,15]. Therefore, the axles of trailers and semi-trailers are one of the main and most important elements that carry the greatest loads during the transport of goods. The axles of semi-trailers are a structural element that withstands both the full weight of the semi-trailer and the load, as well as the reactions of the road surface. Proper construction of the vehicle and its performance in accordance with the requirements of the vehicle approval are key factors in ensuring the durability and reliability of the semi-trailer. Even if these requirements are met, there are cases where the vehicle is no longer used due to structural damage. Due to the measurable costs of decommissioning the vehicle and the threat to traffic safety, each case of damage should be investigated. This is the only way to identify the causes of damage and reduce transport costs.

Potential damage, whatever its cause, is modeled and estimated in modeling. The finite element method (FEM), sometimes referred to as finite element analysis (FEA), is widely used in the literature [16,17,18,19,20,21] to achieve essentially two purposes. One of the aims of this study is to analyze the stresses of the current semi-trailer design or to optimize its design. The other concerns the identification and verification of possible causes of injury. FEM is a computational technique used to obtain approximate solutions to limit value problems in engineering. In other words, a boundary value problem is a mathematical problem where one or more dependent variables must satisfy a differential equation in a known domain of independent variables and satisfy the specific boundary conditions of the domain. Simply put, a model of the object under study is created, it is divided into finite elements, the forces acting on the object are evaluated and the displacements, stresses, reactions and deformations of the elements are calculated. When performing FEM strength calculations, the main task is to calculate the load forces and torques and to model the road conditions so that the numerically modeled conditions reflect the real road conditions [17,22,23,24,25].

During operation, any trailer is subjected to loads that cause stresses, vibrations and noise in various parts of its structure. In order to withstand these loads, the strength, stiffness and fatigue properties of the respective components are required. In addition, the quality of the trailer as a system, which includes energy efficiency, safety and consumer comfort, is highly desirable [23].

Virtually every trailer and semi-trailer has a built-in data collection “black box”. These data are very important in the event of a truck accident. A black box is a general term that can refer to several different elements of computerized systems typically installed in a commercial motor vehicle. In the specific case under analysis, the semi-trailer has an operational data logger integrated in the TEBS modulator, called ODR-Tracker. The ODR-Tracker program records the conditions under which the trailer was operated. These data are used to analyze the use of the vehicle and to evaluate the towing vehicle. During the analysis, it is possible to see the total mileage of the semi-trailer, the number of trips, the working hours of the driver, and so on. Importantly, this program records the average and maximum or exceeded driving speed of the semi-trailer, the air pressure, the frequency of the brake pedal, the braking time and force, the number of actuations of the ABS system and many other parameters [26]. Due to these advantages, the data obtained from this software are often the basis for assessing the efficiency of vehicle use (usually published) and real values of units and part load (most often unpublished). The last mentioned parameter is crucial if it is necessary to recreate the actual operating conditions under which the vehicle components have been damaged.

Very often the literature sources are limited to the analysis of FEM, but no less important is the real analysis of the axis design. When designing and manufacturing semi-trailer axles and other fasteners, it is very important to choose the right design solution. It is also important to choose the right materials from which the structure will be made. Properly designed constructions do not require technological adjustments and are durable. However, in the event of repeated axle fractures of semi-trailers, it is necessary to analyze the structural components, cross-sections and assess the reliability of the overall system [14,24,27,28].

The subject of the research presented in this paper was a damaged axle of a truck semi-trailer. The need to conduct the test arose from the fact that the transport company had several vehicles of the same type, all intensively used, and it was not the first case of damage of this character. Due to the fact that access to the ODR-Tracker data was obtained for the tested case of axle damage, it was decided to use them for analytical calculations and simulations. Thus, not only the geometry of the elements might be mapped, but also the values of the actual load. At the same time, the need to rely solely on nominal data was avoided, which distinguishes the approach presented here from those previously published.

## 2. Materials and Methods

The object of the research presented in this paper was the axle of a semi-trailer damaged during the operation of the vehicle. The damage to its material occurred in an unusual place. This did not happen near the wheel of the vehicle, where the greatest stresses due to road input seem to occur, but at the rocker arm, towards the centre of the vehicle. Therefore, it was decided to look for structural, technological, operational and dynamic factors that could explain the damage in this particular place.

The analysis of the structure and possible defects in the technology of making the elements according to the state after the damage was carried out using macroscopic metallography methods. The vehicle load and traction conditions were checked on the basis of recorded data on the courses of transport. Simulations of the axis dynamics were performed using FEM and based on analytical calculations. All test results complement each other and allow us to confirm or exclude possible causes of cracks.

As the damage to the axle assembly occurred in an unusual place, we decided to start the analysis by checking the frequency and form of natural vibrations. Although the mere occurrence of vibrations does not have to be the cause of damage in the joint, the fatigue strength of the materials is lower than the static one and the number of cycles that can be transferred is always limited.

### 2.1. Finite Element Method Simulations

**Figure 1:**
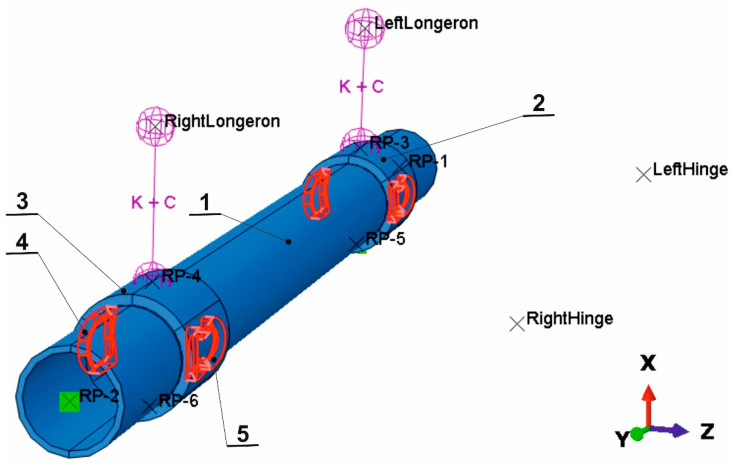
Assembly of the axle with constraint points on longerons and hinges: 1—axis; 2, 3—bushings; 4, 5—angle welds. RightLongeron, LeftLongeron, RightHinge and LeftHinge points have a rotational degree of freedom round the *Y* axis only. Between points RightHinge and RP-4, as well as RP-6, is rigid body type connection to the wishbone and a similar part is in model at the left side. Vehicle front at right.

Numerical simulations were carried out to check the location of potential stress concentration points. The model described in publication [29] was used for the simulation (Figure 1). The axis model (tube) contains 10,120 elements (20,424 nodes) type C3D4R with reduced integration. The mesh elements of the tube were intentionally denser in the area of their interaction with both bushing parts. A similar number of elements (9528) and nodes (15,052) consist of two bushings. For both parts, the mesh of C3D4R elements had been refined at the area of contact with the welds. Welds (for tying bushings and tube) were meshed with C3D4 elements (at least 10,785). The wishbones were modelled as a rigid body (not shown in Figure 1) between points (LeftHinge and RightHinge) and appropriate bushing.

Due to the complex system of interactions (tie, coupling, rigid body) between the geometry elements, the modelled parts were divided into partitions. The largest number of partitions, 16, were separated in the axis part. The quality of the mesh of all parts was checked in terms of the occurrence of geometrical errors, the time of a stable time increment and the consistency of the simulation results with the analytically calculated values.

The stiffness of the linear spring elements installed between axle (RP-3, RP-4) and left and right longerons was taken equal to 4.5 kN/mm. Shock absorbers with a damping coefficient of 10 N s/mm each were included between the same nodes like springs. The model omitted the stiffness and damping of wheels with tires. The wheel weight of 120 kg was simulated as hooked at the axle ends in points RP-1 and RP-2. Additionally, points RP-5 and RP-6 were assigned a mass of 25 kg each, which resulted from taking into account this part of the mass of the spring, the shock absorber and the wishbone, which are displaced together with the solid elements visible in Figure 1. The quality of the mesh of all parts was verified in terms of the occurrence of geometrical errors, the stable time increment and the consistency of the simulation results with the analytically calculated values. Simulations were performed using the Abaqus Explicit software.

The numerical model prepared in the described way was used to analyze the stress distribution in the axle material. The location of the zone of greatest stresses and their values were analyzed in terms of the possibility of damage observed on the real object. Additionally, the model was used to check the mode and frequency of the natural vibrations of the axis. This analysis was used to verify the values taken for the simulation and to check whether the generation of vibrations was related to the load on the axle material in the area of cooperation with the sleeve, where the origin of fracture usually takes place.

### 2.2. Semi-Trailer ODR-Tracker Data Analysis

Trip data is recorded in the ODR-Tracker in the TEBS modulator of the semi-trailer and stores the last 200 trip data. Recording starts when the distance traveled is at least 5 km and the vehicle speed is at least 30 km/h.

According to the Register of Legislation, speed limiters for freight transport with a gross vehicle weight of more than 12 Mg must be set at a maximum speed of 85 km/h.

**Figure 2:**
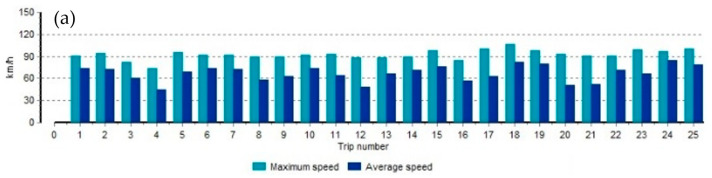

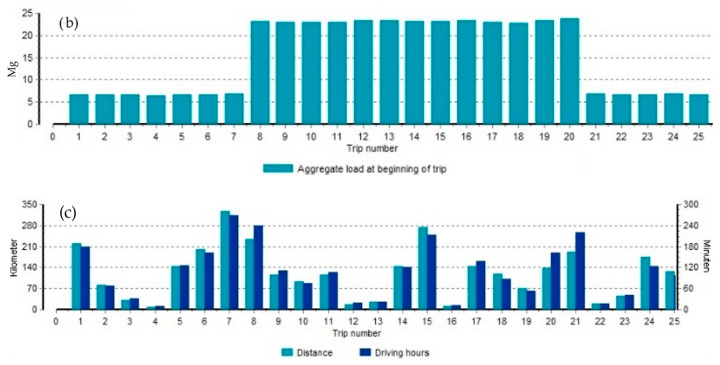
Characteristics of transportation parameters: speed (**a**), load (**b**), distance and time (**c**).

In Figure 2, we can see that the maximum speed was exceeded during almost every trip, with a speed of 90–110 km/h. In the load chart, we see journeys when the semi-trailer was running unloaded or with a small load and journeys when the semi-trailer was fully loaded. Comparing the speed and semi-trailer load diagrams, we see that the speeds were exceeded even when the semi-trailer was fully loaded.

In the event of road irregularities or potholes, a fully loaded semi-trailer is more likely to break under chassis components or components at maximum or close speeds.

We observe that all the numerical values in red are the limits of the set parameters (Table 1). Data in the table are ordered in max speed. In 35% of all trips, we see speeding and exceeding the maximum load on the axle. These are factors that can affect the critical deformations of the axles.

A jump in air pressure gauges has been recorded. Such significant changes in the parameters of the air system (2 times higher than the permissible ones) are possible only in the presence of strong, short-term effects of external factors, such as the wheel entering a sufficiently deep pit or entering an obstacle [21,30]. We can conclude that there were such externalities [24].

### 2.3. Analytical Calculations of an Axle Load

Analytical calculations were carried out for the middle part of the connection of the axle with the sleeve, in the place where the cross-section is the smallest. The geometric moments of inertia of the cross-section, axials and polar were determined. The axle load was assumed on the basis of data published in the literature. The location of the most loaded cross-section and the values of normal and shear stresses were determined. The calculations were carried out when driving on a good quality road and on a bumpy road.

## 3. Results and Discussion

### 3.1. Effects of Numerical Simulations

**Figure 3:**
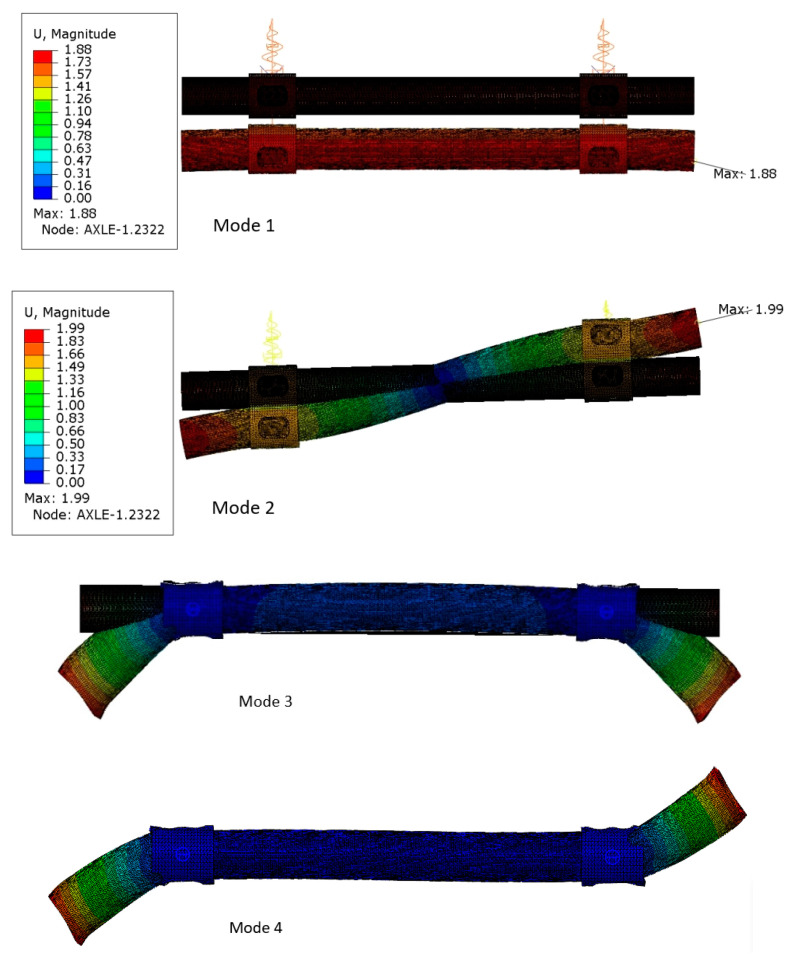
Modes of natural vibrations of the axle. Modes 1 and 2—view from the back side, modes 3 and 4—top view. Deformation scale factor 130.

Mode 1 and 2 take place in a vertical plane (Figure 3). The first mode of vibration has the character of uniform deflection of the complete axis. In addition to the mass of the axle, the frequency of the vibrations is therefore affected by the stiffness of the suspension spring elements. At an excitation frequency of 24 Hz, with a deflection of less than 2 mm, the entire axis vibrates in the vertical direction. The figure shows the same deflection of the suspension springs for mode 1. For this reason, this mode of vibration cannot generate stresses in the material. The vibration period in this case is about 41 ms. Therefore, if the vehicle was moving at a speed of 90 km/h (a value close to the maximum speed in Figure 1), this mode of vibration would occur when the wheel passes over bumps on the road spaced approximately every 1 m.

**Figure 4:**
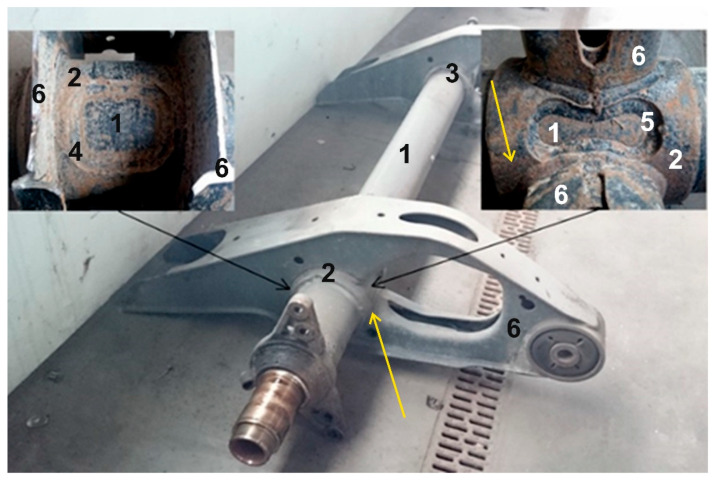
Mounting points for brackets and couplings: 1—axis; 2, 3—bushings; 4, 5—welds; 6—arm. The fracture origin area is pointed out by yellow arrows.

The second mode of vibration also depends on the stiffness of the elastic elements of the suspension. Unlike the first form, this one involves the generation of stresses in the material, because the right and left sides of the axis deform in opposite directions—up and down. The greatest deflection, about 2 mm, should be expected at the end of the axle, where the wheels are mounted. This mode of vibration can occur at an excitation frequency of about 56 Hz, which is more than twice the first one. The greatest stresses should be generated in the area of the smallest deflection, i.e., about half the length of the axle, as well as from the outside, where the axle and bushing are connected by a weld cooperate. It may be concluded that vibrations of this type will not have a significant impact on the fatigue load of the central part of the axle, where good quality protective coating was observed. A greater risk may occur in the area of axle and bushing contact. Due to the impact of sand, stones and water thrown by the wheels, the protective coating is subject to faster wear there (see Figure 4). In addition, the mutual cooperation of the two parts that make up the axle may cause abrasion of the paint coating even in places invisible from the outside.

For the second form of vibration to occur, the excitation existing on the road should be approximately every 443 mm, if the vehicle is moving at a speed of 90 km/h. This distance is shorter than the dynamic radius of the wheel (about 497 mm), so in addition to the elastic characteristics of the suspension, the stiffness of the tires would be important here; this was neglected in the simulation.

The first form of vibration is the most likely, i.e., it can most often occur when the vehicle is moving at a speed of 90 km/h.

Mode 3 and mode 4 (Figure 3) are only in a horizontal plane. In this case, the maximum axle deflection of 2.16 mm occurs only on the outside of the wishbones. The excitation frequency necessary to induce these vibration modes is about 149 Hz, which is almost 3 times higher than for mode 2. The occurrence of such an excitation frequency is unlikely under operating conditions. The difference in the natural frequency for modes 3 and 4 does not exceed 0.32 Hz, which is less than 3%.

None of the four modes of vibration described can be caused by forces from rotating suspension components. In a vehicle traveling at a speed of 90 km/h, a wheel with a dynamic radius of 497 mm rotates without slipping at a frequency of less than 6 Hz. So, it is much lower than the frequency 24 Hz calculated for mode 1.

### 3.2. Axle Construction Analysis

The test object consists of round profile axle, couplings with welded brackets, shock absorbers, airbags, rubber bushings, wheel hubs with disc brake system and other fasteners. Mounting points for brackets and axle couplings are shown in Figure 4.

**Figure 5:**
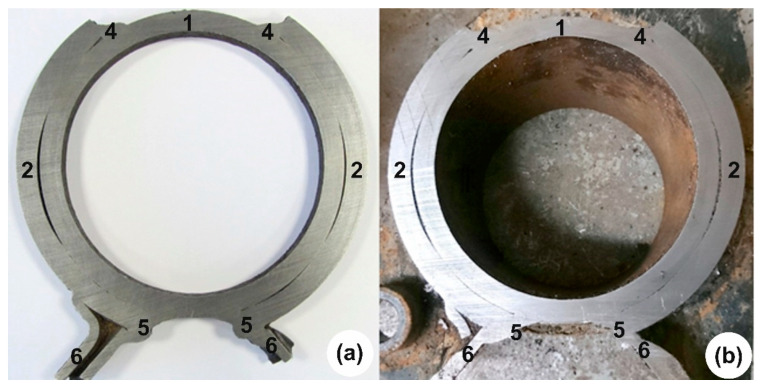
Two sides of the same cross-section at welds of the axis unit: (**a**)—broken side; (**b**)—unbroken side. The numerals mean: 1—axis; 2—bushing; 4, 5—angle welds; 6—arm.

**Figure 6:**
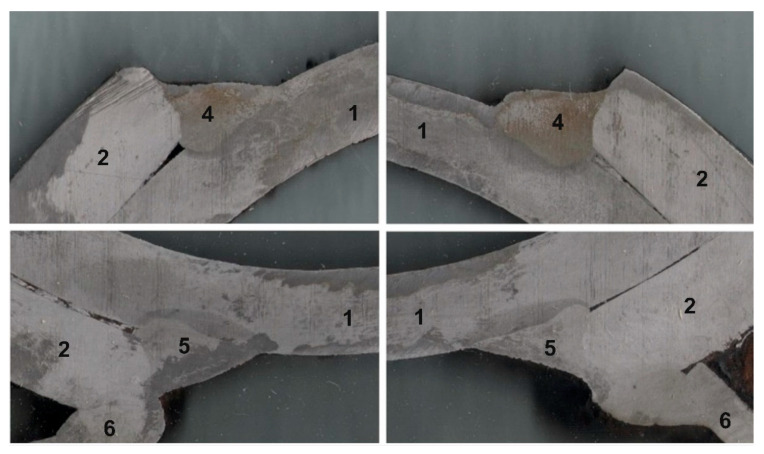
Cross-section of angle welds: 1—axis; 2—bushing; 4, 5—angle welds; 6—arm.

The brackets are connected to the shaft with sleeve joints with no thermal effect and are welded in four places. In the coupling joint, two technological holes of different geometries are cut from opposite sides. In those places, the couplings are welded to the shaft (Figure 5). From the results, we can see that the geometry of the shaft and the coupling changes are due to the welding process (there is a gap between the shaft and the coupling) (Figure 6).

Examining the fastening of the brackets and couplings (Figure 4) we see that the total area of contact of the coupling with the axis is 84,811 mm^2^. The area of the welds on which the sleeve joint with the shaft is reinforced is 8628 mm^2^. During the destructive studies and the examination of the fracture (Figure 5), we notice that the contact area has changed due to welding. The coupling is detached from the axis and we can say that the axis is “held” only by the welds, which make up only 10% of the total contact area in the project [31,32,33].

We see that there is a gap between the shaft and the coupling connection, in places up to 1.5 mm (Figure 5a). To make sure that this is not the result of a fracture, but a construction, we also cut the part of the shaft without the fracture. In Figure 5b), we see that there is also a gap in the cross-section of the fractured side of the shaft between the shaft and the coupling joint. This confirms the assumption that the gap is not a consequence of a fracture [34].

The measurement of the welded joints shall assess the conformity of the joints, their arrangement and the amounts of defects observed during the visual inspection: inclusions, accumulations, cuts, indentations, convexities or depressions in the seam surface, fractures of the seam surface size and deviations in geometrical parameters.

Seam defects and damage are identified according to [35,36] of the literature.

The integrity of the weld seam was inspected and no cracks were observed. After the section of the axle at the welded joints (Figure 6), the cross-sections of the welds were inspected. Defects observed: 402—Non-weld (insufficient weld), 500—Shape defects (irregular geometry of the weld, imperfect shape of the outer surface of the weld, different geometries of the cross-sectional area, cross-sectional areas of the weld vary from 6 to 20%), 505—Insufficient weld edges, 509—Depression (formed by gravity; the amount of the weld metal on the axle metal is higher than on the coupling metal; significantly greater thermal impact area), large angular seam asymmetry (excessive structural unevenness), 513—Uneven width (large seam width tolerances), 514—Uneven surface, 602—Splashes (added metal droplets spattered by welding and adhering to the base and hardened seam metal).

For welded joints, the area of thermal exposure close to the weld limit is of great importance. The range of thermal effects of the test seam is very different: from 1 mm to 3.5 mm. The metal heats the seam almost to the melting point. At that point, the metal is overheated, so this area usually determines the strength of the welded joint.

It can be seen that there is a significant difference in the load on the axle material depending on whether the considered point is located on the outside of the wishbone (between the wishbone and the wheel hub journal) or in the place between the wishbones. During driving and acceleration, if friction in rolling bearings is omitted, no torsional stresses are generated in this section of the axle which is closer to the wheel. The torsional load of this part of the axle occurs only during braking. Apart from that, bending stresses prevail; the source of which is the weight on one side of the axle (normally half of the whole axle load), and geometric extortions caused by unevenness in the road [37,38]. In the middle part of the axle, between the wishbones, bending and torsion loads occur simultaneously. In addition, this load is greater than the difference in the deflection (rotation) of the wishbones of the same axle.

In the case of analytical calculations, which are shown below, all mentioned load types are included.

### 3.3. Analytical Calculations

#### 3.3.1. The Moments of Inertia of the Cross-Section

**Figure 7:**
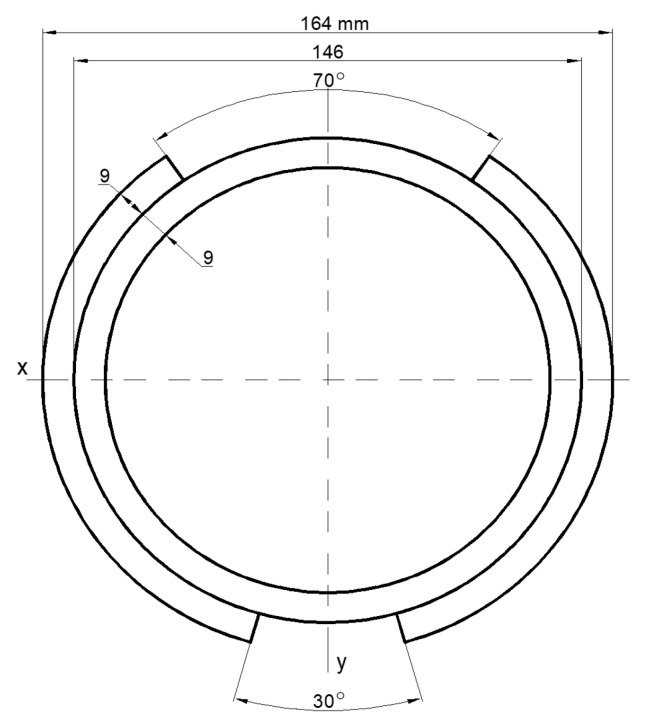
Axis cross-section geometry for solution of moments of inertia.

The geometry of the cross-section is calculated as a complex figure consisting of an annular tube with two external brackets (Figure 7). Additional areas in the cross-section of the welds and corbels (Figure 5) are unvalued. The moments of inertia of the cross-section of the axle are determined as a sum of the central annular tube and the two external brackets.

The shaft is deformed by two bending moments and torque and so, two moments of inertia and a polar moment of inertia are determined.

The polar moment of inertia in general cases [39] is:

(1) $$I_{p}=\iint_{A}^{}\rho^{2}dA=\iint_{A}^{}\rho^{2}\rho \;d\varphi d\rho$$

**Figure 8:**
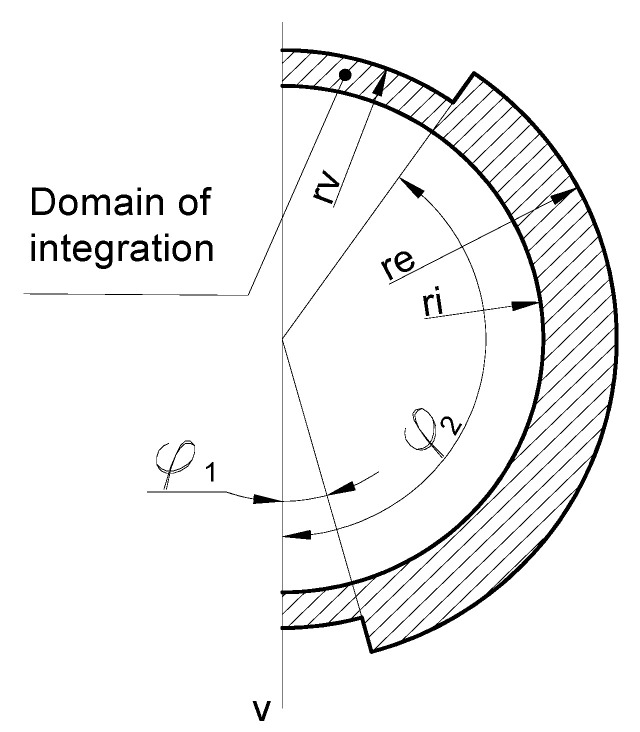
Domain of integration.

Due to symmetry, only half of the area is used as a domain of integration (Figure 8) for a solution of the polar moment of inertia.

Using integration Formula (1) for a defined domain (Figure 8) from vertical axis *v*:

(2) $$I_{p}=\int_{0}^{\varphi_{1}}d\varphi \int_{r_{i}}^{r_{v}}\rho^{3}\;d\rho +\int_{\varphi_{1}}^{\varphi_{2}}d\varphi \int_{r_{i}}^{r_{e}}\rho^{3}\;d\rho +\int_{\varphi_{2}}^{\pi }d\varphi \int_{r_{i}}^{r_{v}}\rho^{3}\;d\rho \;,$$

In cases when (according to Figure 7) values of particular angles and radiuses are equal:

(3) $$\varphi_{1}=\frac{30{}^{\circ}}{2}=15{}^{\circ}\;;\;\varphi_{2}=180{}^{\circ}-\frac{70{}^{\circ}}{2}=145{}^{\circ},$$

(4) $$r_{i}=\frac{146\;\mathrm{mm}-2*9\;\mathrm{mm}}{2}=64\;\mathrm{mm}\;;\;r_{v}=\frac{146\;\mathrm{mm}}{2}=73\;\mathrm{mm}\;;\;r_{v}=\frac{164\;\mathrm{mm}}{2}=82\;\mathrm{mm}\;,$$

The polar moment of inertia $I_{p}\;$ is equal $37.3\cdot 10^{6}\;{\mathrm{mm}}^{4}$.

**Figure 9:**
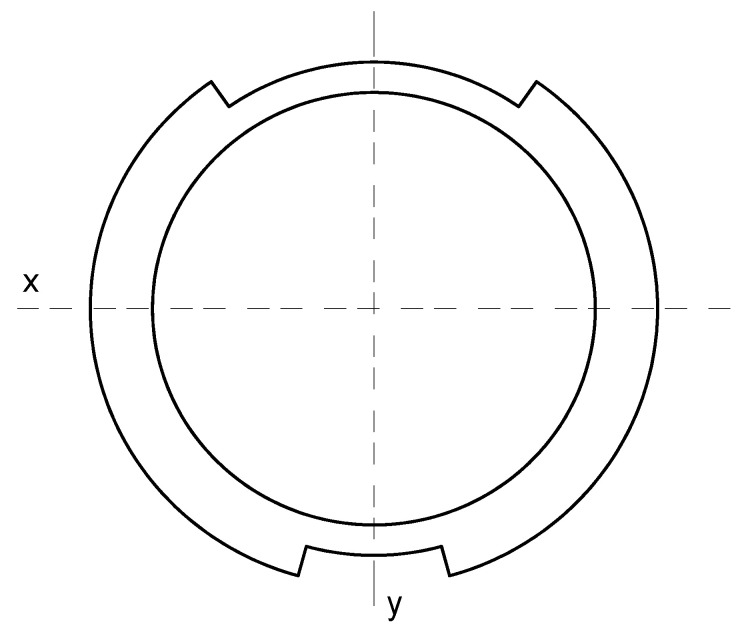
Area for determine moments of inertia.

The area of the cross-section and moments of inertia around the x and y axes (Figure 9) are solved and checked with CAD program.

The geometrical parameters of cross-section: area *A* = 7030 mm^4^; moments of inertia: $I_{x}=15.6\cdot 10^{6}{\;\mathrm{mm}}^{4}$; $I_{y}=21.7\cdot 10^{6}{\;\mathrm{mm}}^{4}$.

**Figure 10:**
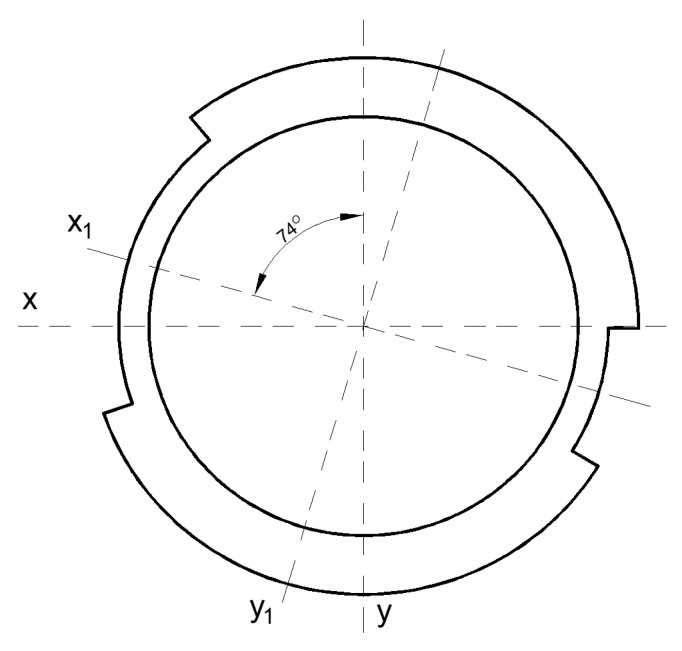
Area to determine moments of inertia with rotation.

The real orientation of the cross-section of the axle is rotated with an angle of 74 degrees (Figure 10).

The moments of inertia around the horizontal and vertical axis: $I_{x1}=21.2\cdot 10^{6}{\;\mathrm{mm}}^{4}$; $I_{y1}=16.2\cdot 10^{6}\;{\mathrm{mm}}^{4}$. The polar moment of inertia and section modules will be used to determine maximal stresses in the cross-section and the finding of critical points.

#### 3.3.2. Analytical Normal and Shear Stresses in Dangerous Zone of Cross-Section

**Figure 11:**
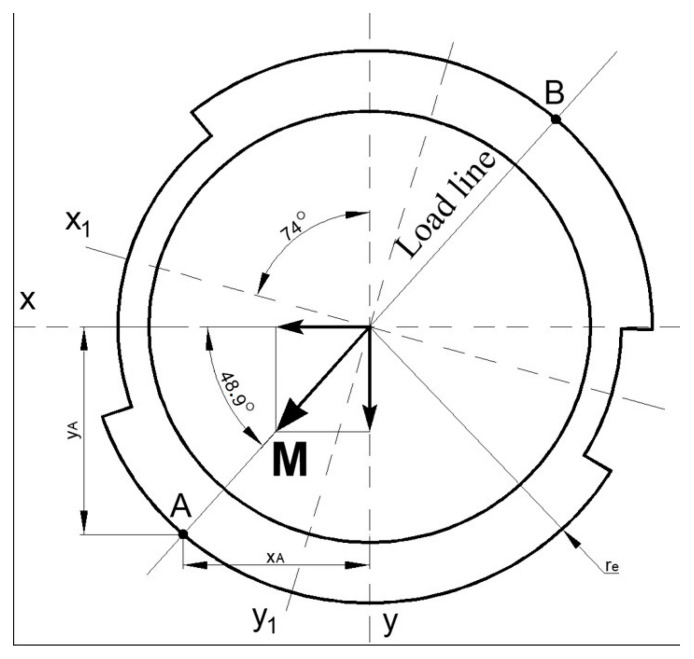
Sum moments and load line direction.

Bending and torsion are described in [40,41] and the internal forces of the axle are taken from [42] and will be used in this chapter. The investigation quarter of the cross-section is in left and down with both positive stresses due to bending moments (Figure 11). In this quarter exists welding of internal and external tubes; consequently, welding is a stress concentrator factor. It follows that critical point A with maximal stresses is in the dangerous zone of weld or near this zone.

Two types of heavy loadings of axle are used to determine the stresses in point A [21,39,40,41]:

1. Driving of the truck on highways without appreciable path holes (the maximal depth is 50 mm);
2. Diving of the truck on highways or roads with path holes with a depth of up to 100 mm.

#### 3.3.3. Driving of the Truck on Highways

Internal forces from [29] are:

- Bending moment *M_x_* = 31.1 kNm;
- Bending moment *M_y_* = 27.3 kNm;
- Torque *T* = 9.0 kNm.

Both bending moments can be replaced with resultant vector sum **M** (Figure 11). This sum moment is acting on a plane which is termed as a load plane. The intersection of the load plane with the cross-sectional plane is the load line.

The direction of the load line is described by angle *φ*:

(5) $$\varphi =\mathrm{atan}\left(\frac{M_{x}}{M_{y}}\right)=\mathrm{atan}\left(\frac{31.1\;\mathrm{kNm}}{27.3\;\mathrm{kNm}}\right)=0.85\;\mathrm{rad}=48.7^{{}^{\circ}}$$

Points A and B are on the plane where sum moment **M** acting and normal stresses are extreme. From previous investigations [39], the position of the dangerous quarter has been found in the cross-section—front and down quarter. As a result, the normal stresses in point A are determined:

(6) $$\sigma_{A}=\frac{M_{x}}{I_{x1}}\cdot y_{A}+\frac{M_{y}}{I_{y1}}\cdot x_{A},$$

where, *y_A_* and *x_A_* are coordinates of the point A in Figure 11.

The real values of coordinates are:

$$x_{A}=r_{e}\cdot cos\varphi =82\cdot cos48.7^{{}^{\circ}}=54.1\;\mathrm{mm}$$

$$y_{A}=r_{e}\cdot sin\varphi =82\cdot sin48.7^{{}^{\circ}}=61.6\;\mathrm{mm}$$

The normal stress in point A due to bending moments acting about the horizontal and vertical axes is:

(7) $$\sigma_{A}=\frac{31.1\cdot 10^{6}\;\mathrm{Nmm}}{21.2\cdot 10^{-6\;}\;\mathrm{Nmm}}\cdot 61.6\;\mathrm{mm}+\frac{27.3\cdot 10^{6}\;\mathrm{Nmm}}{16.2\cdot 10^{-6}\;\mathrm{Nmm}}\cdot 54.1\;\mathrm{mm}=161\;\mathrm{MPa}$$

Shearing stresses in point A:

(8) $$\tau_{A}=\frac{T}{I_{p}}\cdot \rho_{A}=\frac{9.00\cdot 10^{6}\;\mathrm{Nmm}}{37.3\cdot 10^{6}\;{\mathrm{mm}}^{4}}\cdot 82\;\mathrm{mm}=19.8\;\mathrm{MPa}$$

Compound influence of bending and torsion is calculated below:

(9) $$\sigma_{d,A}=\sqrt{\sigma_{A}^{2}+3\cdot \tau_{A}^{2}}=\sqrt{161^{2}\;\mathrm{MPa}+3\cdot 19.8^{2}\;\mathrm{MPa}}=165\;\mathrm{MPa}$$

The value of stress when the truck drives on highways proves that the construction is enough strong. The dominant type of stress here is normal and its value is less than 30 % of the yield strength (581 MPa) for axis material.

#### 3.3.4. Driving of the Truck on Roads with Roughness

For analyzing the case of driving of the track on pavement roads, values of loading forces of the axle was taken from publication [29]. They are:

- Axial force caused, e.g., by truck turning *N* = 44.1 kN;
- Bending moment which is generated by the force acting between the tire and the road surface *M_x_* = 53.3 kNm and *M_y_* = 27.3 kNm;
- The torque of the axle which is a sum of the braking force acting at the dynamic radius of the tire *T* = 12.4 kNm.

**Figure 12:**
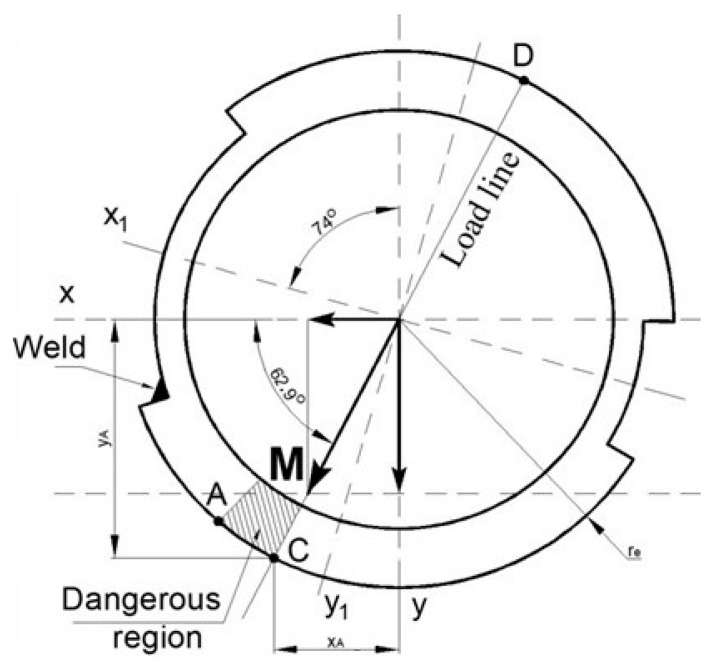
Load line direction and rotation of coordination system.

The load line changed direction due to increasing the bending moment about the *x* axis (Figure 12). The angle of the moment vector was calculated according to the equation:

(10) $$\varphi =\mathrm{atan}\left(\frac{M_{x}}{M_{y}}\right)=\mathrm{atan}\left(\frac{53.3\;\mathrm{kNm}}{27.3\;\mathrm{kNm}}\right)=1.098\;\mathrm{rad}=62.9^{{}^{\circ}}$$

The normal stresses have additional influence due to the axial force increasing due to bending moments and expressed as follows:

(11) $$\sigma_{C}=\frac{N}{A}+\frac{M_{x}}{I_{x}}\cdot y_{C}+\frac{M_{y}}{I_{y}}\cdot x_{C}$$

The real values of coordinates are:

$$x_{C}=r_{e}\cdot cos\varphi =82\cdot cos62.9^{{}^{\circ}}=37.4\;\mathrm{mm}$$

$$y_{C}=r_{e}\cdot sin\varphi =82\cdot sin62.9^{{}^{\circ}}=73.0\;\mathrm{mm}$$

With values:

(12) $$\sigma_{C}=\frac{44.1\cdot 10^{3}\;N}{7030{\;\mathrm{mm}}^{2}}+\frac{53.3\cdot 10^{6}\;\mathrm{Nmm}}{21.2\cdot 10^{6}{\;\mathrm{mm}}^{4}}\cdot 73\;\mathrm{mm}+\frac{27.3\cdot 10^{6}\;\mathrm{Nmm}}{16.2\cdot 10^{6}{\;\mathrm{mm}}^{4}}\cdot 37.4\;\mathrm{mm}=253\;\mathrm{MPa}$$

Shearing stresses with road roughness influence:

(13) $$\tau_{C}=\frac{T}{I_{p}}\cdot r_{e}=\frac{12.4\cdot 10^{9}\;\mathrm{Nmm}}{37.3\cdot 10^{6}\;{\mathrm{mm}}^{4}}\cdot 82\;\mathrm{mm}=4.05\;\mathrm{MPa}$$

Compound determining stress in weld region:

(14) $$\sigma_{d,C}=\sqrt{\sigma_{C}^{2}+3\cdot \tau_{C}^{2}}=\sqrt{253^{2}+3\cdot 4.05^{2}}=253\;\mathrm{MPa}$$

Advancing load strength determined by the dynamics coefficient 1.2 [35,42,43,44].

On highways, the maximal stress in a dangerous zone will increase due to a stress concentrator: from *σ*_*d*,*A*_ = 161·1.2 = 193 MPa, until *σ*_*d*,*A*_ = 165·1.2 = 198 MPa.

During driving on low quality roads, the actual stresses in the axle increase to: *σ*_*d*,*C*_ = 253·1.2 = 304 MPa.

Between points A and C, there is a dangerous region of cross-sections with extreme possible stresses. This region has a stable position in plane due to bending moments. When the wheel meets roughness in the road, due to the rotating of the cross-section counter clockwise, the weld between the external and internal tubes with the entire section also rotates to a dangerous zone (Figure 12).

Another stress concentrator factor is weld in the cross-section. Since the position of the weld seam is different, such as critical points with extreme stresses in the load line, the stresses in the weld cross-sectional area will be analyzed in the next paper.

#### 3.3.5. Fatigue of Coupling Node

In order to specify a strength for axle under repetitive loading, it is necessary to determine fatigue limit $\sigma_{-1}$ for steel of axle. It could be performed experimentally and theoretically. Endurance experiments are expensive and need special laboratory equipment.

In theory [36], fatigue limit $\sigma_{-1}\;$ for the symmetrical cycle described as function on ultimate tensile stress $\sigma_{u}$. For tension, this function is $\sigma_{-1}=0.28\cdot \sigma_{u}$, for bending $\sigma_{-1}=0.40\cdot \sigma_{u}$, respectively, for torsion $\tau_{-1}=0.22\cdot \sigma_{u}$. Stresses of axle vary according to non-symmetrical function, depend on road roughness and could be similar due to distribution in Figure 12.

Is it known that non symmetrical cycle loading has a lesser endurance limit [45]. It is enough to know that for those analyses, that in bending and torsion fatigue limit is maximum 50% of ultimate limit. From previous investigations [29,42], it is known that ultimate tensile stress is 663 MPa, so a theoretical maximal fatigue limit of steel will not exceed 332 MPa; accordingly, this limit will be decreased in reality. If the value of stresses generated in the material of the vehicle axle (304 MPa) increased by about 9%, they would reach the calculated tensile stress limit at fatigue load.

It can be seen that the second term of the sum in Equation (12) has the greatest influence on the stress value. With unchanged values of other parameters, a 20-degree left rotation of the system shown in Figure 12 could cause such an increase in stresses that they would reach the fatigue stress limit. The same effect could be caused by changing the direction of action of the unchanged value of the total moment M by about 20 degrees to the right. In this case, the angle would not be 62.8 but 42.8 degrees.

It is worth paying attention to an additional aspect, structural or technological. It was shown that the elements of the tested axle did not touch each other over the maximum surface area. This means that the entire cross-section could not be active in the load transfer. When driving on a poor quality road in this case, damage may occur in the observed place. In the literature [5], one can find an example of simulating a similar system of the whole vehicle in which the authors indicate the same area as having a critically low fatigue strength.

## 4. Conclusions

1. From analyzing the ODR-Tracker data, it was found that 35% of all trips were speeding and exceeding the maximum axle load.
2. In destructive studies, it was observed that the contact area between the two elements changed due to welding. The coupling is in contact with the shaft only at the points where the welds are located, which is only 10% of the total contact area of the joint.
3. The measurement of welded joints shall assess the conformity of the joints, their arrangement and the amounts of defects observed during the visual inspection: inclusions, accumulations, cuts, indentations, convexities or depressions in the seam surface, fractures of the seam surface size and deviations in geometrical parameters.
4. After the section of the axle at the welded joints, the cross-sections of the welds were inspected. Defects observed: non-weld, shape defects, insufficient weld edges, depression, large angular seam asymmetry, uneven width, uneven surface and splashes.
5. Maximal analytical stresses were calculated in the dangerous region for two types of driving. On highways stresses in coupling, the node critical point increase until 198 MPa, on roads with roughness—304 MPa. Overload by 9% would cause a fatigue limit of steel.
6. The extreme stresses occur in the defined dangerous region of the cross-section and in rotating the cross-section, the weld is closer to this region with increasing value of stresses.
7. It is shown, that in the dangerous quarter of the cross-section exists weld and the weld position does not coincide with the load line. Due to welding, additional stress concentration in the weld region appears and results from this investigation will be used for weld structure analyses.
8. The first vibration mode takes place at the excitation frequency of 24 Hz and can most often occur when the vehicle is moving at a speed of 90 km/h. However, the deformation of the system for this mode is such that it does not generate stresses in the material. The deformation of the axis in the hazardous area, where the damage was observed, may occur as a result of natural vibrations of the second mode. The excitation frequency in this case would have to be 56 Hz, which is less likely under operating conditions.

## Figures and Tables

**Table 1:** Vehicle parameters registered during full-loaded transport.

Distance, km	Driving Time, h	Max Speed, km/h	Pressure, MPa	Break		Max Load, Mg
				Actuations, -	Frequency, 1/km	
10.9	00:12	78	**0.285**	2	0.18	24.1
10.9	00:11	85	**0.410**	2	0.18	24.1
49.7	01:10	85	0.200	44	0.89	24.1
16.5	00:20	88	**0.225**	12	0.73	24.1
234.2	04:00	89	0.195	117	0.50	24.1
116.3	01:51	90	0.185	52	0.45	24.1
68.7	00:56	92	**0.270**	20	0.29	25.1
34.1	00:37	92	0.155	37	1.09	24.1
116.0	01:47	93	**0.205**	43	0.37	24.1
137.9	02:41	93	0.165	83	0.60	24.1
274.3	03:41	94	0.175	76	0.28	24.6
197.0	02:45	95	**0.240**	59	0.30	25.6
132.9	01:19	97	**0.210**	14	0.11	24.1
262.3	03:59	97	0.180	180	0.69	24.6
70.5	00:53	98	0.185	22	0.31	24.1
17.9	00:17	99	0.190	12	0.67	24.1
109.7	01:33	101	**0.215**	45	0.41	24.1
179.0	02:35	101	0.195	85	0.47	24.1
258.1	03:08	101	0.185	94	0.36	24.1

## Data Availability

Not applicable.
